# Pathways to malaria persistence in remote central Vietnam: a mixed-method study of health care and the community

**DOI:** 10.1186/1471-2458-9-85

**Published:** 2009-03-23

**Authors:** Martha Morrow, Quy A Nguyen, Sonia Caruana, Beverley A Biggs, Nhan H Doan, Tien T Nong

**Affiliations:** 1 Nossal Institute for Global Health, University of Melbourne, Melbourne, Vic 3010, Australia; 2 National Institute of Malariology, Parasitology and Entomology, BC 10200 Tu Liem, Hanoi, Vietnam; 3 Department of Medicine, University of Melbourne, Melbourne, Vic 3010, Australia

## Abstract

**Background:**

There is increasing interest in underlying socio-cultural, economic, environmental and health-system influences on the persistence of malaria. Vietnam is a Mekong regional 'success story' after dramatic declines in malaria incidence following introduction of a national control program providing free bed-nets, diagnosis and treatment. Malaria has largely retreated to pockets near international borders in central Vietnam, where it remains a burden particularly among impoverished ethnic minorities. In these areas commune and village health workers are lynchpins of the program. This study in the central province of Quang Tri aimed to contribute to more effective malaria control in Vietnam by documenting the *non-biological *pathways to malaria persistence in two districts.

**Methods:**

Multiple and mixed (qualitative and quantitative) methods were used. The formative stage comprised community meetings, observation of bed-net use, and focus group discussions and semi-structured interviews with health managers, providers and community. Formative results were used to guide development of tools for the assessment stage, which included a provider quiz, structured surveys with 160 community members and 16 village health workers, and quality check of microscopy facilities and health records at district and commune levels. Descriptive statistics and chi-square analysis were used for quantitative data.

**Results:**

The study's key findings were the inadequacy of bed-nets (only 45% of households were fully covered) and sub-optimal diagnosis and treatment at local levels. Bed-net insufficiencies were exacerbated by customary sleeping patterns and population mobility. While care at district level seemed good, about a third of patients reportedly self-discharged early and many were lost to follow-up. Commune and village data suggested that approximately half of febrile patients were treated presumptively, and 10 village health workers did not carry artesunate to treat the potentially deadly and common *P. falciparum *malaria. Some staff lacked diagnostic skills, time for duties, and quality microscopy equipment. A few gaps were found in community knowledge and reported behaviours.

**Conclusion:**

Malaria control cannot be achieved through community education alone in this region. Whilst appropriate awareness-raising is needed, it is most urgent to address weaknesses at systems level, including bed-net distribution, health provider staffing and skills, as well as equipment and supplies.

## Background

Malaria remains a major global threat and its control is one of the Millennium Development Goals. Anti-malarial drug resistance, linked to both unnecessary and inadequate drug intake, creates risks for malaria resurgence, and is a major challenge for malaria control [1]. Insecticide-treated bed-nets and effective anti-malarial drug combinations are essential components of control programs [2,3]. However, effective control requires consistent action from both *health systems *and *community*, and an understanding of features that precipitate risk, such as development projects bringing labourers through forested areas [4]. The broad social (i.e. non-biological) aspects of control are thus critical to success [5].

Overextension or poor training of health staff can undermine diagnosis and treatment, while bed-net distribution requires reliable systems that are difficult for impoverished, isolated settings. Patients may not seek care if they lack knowledge or money for treatment or transportation, or may seek care from multiple sources [6-8,3], making implementation of treatment guidelines – and health information systems – problematic [9].

Vietnam is considered a Mekong region malaria 'success story' after the introduction of a National Malaria Control Program (NMCP) in 1991 to address a spike in cases and increasingly widespread drug resistance. The NMCP provided free anti-malarials (especially artemisinin to treat *P. falciparum*, Vietnam's most prevalent – and most deadly if untreated – strain, impregnated bed-nets, twice-yearly home insecticide spraying, and early diagnosis and treatment. Declines from 1.2 million clinical cases (without confirmed blood tests) and 4646 recorded deaths in 1991, to 185 529 clinical cases and 50 deaths in 2002, are attributed to the program [9-12]. Managed by the National Institute for Malariology, Parasitology and Entomology (NIMPE), the NMCP relies on vertical and horizontal collaborations from central to village level.

By 2000 malaria had largely retreated to 84 districts in 15 (out of 61) provinces, with an estimated population of 7.2 million (vs 83 million nationwide). Over 90% of severe cases and deaths occurred in mountainous, forested and largely ethnic minority areas of central Vietnam [9,12], often along international borders, where the common mosquito vectors that transmit malaria, *Anopheles dirus *and *An minimus*, are plentiful [13]. These ethnic minorities tend to be impoverished, poorly educated, culturally and linguistically distinct, and living in dispersed, less accessible settlements; such areas 'represent a real challenge for the [NMCP]' [9] (p.217). Therefore, it is both instructive and timely to investigate persistent malaria in such settings.

In 2002–03 the central province of Quang Tri was among Vietnam's highest malaria burden provinces with 4178 cases (confirmed and clinical). Over 9% of Quang Tri's approximately 573 000 inhabitants are ethnic minorities, overwhelmingly Van Kieu and Paco [14], who live astride the border with Lao PDR in districts with rugged, lush terrain, frequent rainfall, and large infrastructure projects within forests (including a bridge and road along the old Ho Chi Minh Trail). These contextual aspects, together with poverty, low education levels, cross-border mobility, and cultural diversity, made this an appropriate study site for malaria social science research.

This paper reports on a collaborative study aiming to contribute to malaria control in Vietnam by documenting the *non-biological *pathways to malaria persistence in two districts. The objectives were to identify the role and nature of health system and community factors *directly *linked to malaria persistence, and *underlying *influences that help explain the direct factors. The study was undertaken by Vietnamese and Australian researchers from March 2004 to April 2005.

## Methods

In order to meet the study objectives we chose a flexible study design with multiple methods (both qualitative and quantitative). Mixed-method approaches permit explication of complex interrelationships between actors and systems, and have been used for malaria social research [15,16,5]. Data were collected in two stages. The formative stage used mainly qualitative tools to help define and expand thematic areas of enquiry; these data were rapidly reviewed to inform the (mainly quantitative) tools used for the assessment stage. An overview of methods and samples appears in Table 1. NIMPE investigators were trained by Australian colleagues and collected all data during 3 field visits.

**Table 1:** Summary of methods and data sources (both stages)

**Sample/source/focus**	**Location**	**Method (number)**
**FORMATIVE STAGE**		

MC officials, local government, mass organisations, hospitals	2 district capitals	Community Meetings (approx 20 participants each)

Provincial MC officials	Provincial capital	SSIs (2)

District MC secretaries	2 district capitals	SSIs (2)

District Hospital managers	2 district capitals	Informal group discussion (2)

Anti-malarials sold at market	1 border commune	Observation (1)

Village environment	6 villages	Observation (6)

District Hospital staff	2 District Hospitals	SSIs (3)

Commune health staff	6 Commune Health Stations	FGDs (5) SSIs (4)

Village Health Workers	6 communes	FGD (1) SSIs (13 in 5 communes)

Community members	6 communes	FGD (1 with women) SSIs (14 men, 6 women)

**ASSESSMENT STAGE**		

District Hospital staff	2 District Hospitals	Tests (11 open questions) (14)

Malaria patient record cards	2 District Hospitals	Case numbers year-to-date (one DH); Review of previous month's cards DH-A (53) & DH-B (35)

Microscope points	2 District Hospitals & 4 Commune Health Stations	Observation (6)

Patient treatment logs	4 Commune Health Stations	Breakdown of year-to-date case numbers (one CHS); Review of previous 3 months (4 CHSs)

Village Health Workers	16 villages	KAP survey (16) (1/village)

Community members	16 villages	KAP survey (160) (10/village)

Bed-net use and quality	16 villages	Use observed in night-time home visits (55); quality observed in KAP survey home visits (160)

MC = malaria control; FGD = Focus Group Discussion; SSI = Semi-structured Interview; KAP = Knowledge, Attitudes and Practices

### Choice of Study Sites

Among Quang Tri's 8 districts, two (hereafter, A and B) were selected for their greater malaria caseload and proximity to the Lao border. At the 1999 census, district A's population was 54 547 and B's was 27 000; the vast majority were Van Kieu and Paco. For the formative stage we chose 3 border communes per district.

For the assessment phase we used 2 of these communes per district (i.e. total 4 communes) in order to ensure sufficient sample recruitment within the timeframe in view of the low population density and transportation difficulties. From each commune's approx 10 villages we selected 4 with varying ease of access as well as distance from the commune health station (i.e. total 16 villages).

### Development and Use of Instruments, Sampling and Ethics

In the formative stage we held community meetings with district stakeholders to establish rapport and elicit local information and views. Semi-Structured Interviews (SSIs) and Focus Group Discussions (FGDs) using flexible guides were held to explore beliefs, attitudes, awareness, care seeking/providing and circumstances relevant to malaria exposure and control with all provincial and district MC managers and Commune Health Stations (CHS) staff, a convenience sample of VHWs, and community members (village heads and adult men and women, recruited purposively).

For the assessment stage we developed and administered face-to-face structured knowledge, attitudes and practices (KAP) surveys in the 16 villages, one with every Village Health Worker (VHW) (n = 16) and another with10 community members per village (n = 160), respectively. The community sample size was determined on the basis of time, resources and feasibility, along with power to conduct tests of significance on some demographic variables. Sampling was undertaken randomly from village household lists, stratified for equal numbers of men and women aged 18–48. Van Kieu interpreters (one male and one female) were used for nearly all community surveys after training by NIMPE researchers. We also devised observation check-lists to assess visibility and currency of malaria treatment guidelines, quality of CHS microscopy, and bed-net quality during KAP survey home visits. Actual bed-net use was determined by unannounced night visits to 55 homes in 2 communes. To obtain an impression of provider knowledge and guidelines adherence, we quizzed (11 open questions) district hospital (DH) staff involved in malaria control and available on the day, and reviewed one month of patient records from both DHs and 3 months of treatment logs from all 4 CHSs; comprehensive malaria case record numbers for the first 9 months of the year were collated from one DH and one CHS.

Potential participants were assured that participation was voluntary and confidential and refusal would have no negative consequences. As is common in Vietnam, all agreed to participate; verbal informed consent was taken. Participants were given a t-shirt with a malaria control message in appreciation. The study was approved by NIMPE's Human Research Ethics Committee for Medical-Biological Research, and the University of Melbourne's Human Research Ethics Committee. Instruments were developed in English, translated into Vietnamese (and back-translated) and pre-tested with a convenience sample in the study area.

### Data Management and Analysis

Notes were taken during SSIs and FGDs; transcripts were not prepared due to time constraints. Researchers reviewed the formative data to finalise the assessment stage tools. Check-list data, health record reviews and quiz results were collated. KAP survey data were analysed using *Stata *v8.0 (descriptive statistics and chi square tests), and community level differences calculated for location, sex and education. Interpretation of findings was iterative and involved all data sources and researchers; together we distilled a subset of triangulated findings that offered a coherent picture of the interplay between direct and underlying influences on persistent malaria.

## Results

Provincial records showed a continued high malaria burden in Quang Tri in 2004, with a total of 3958 cases (both clinical and slide-confirmed), a slight decline from 2003 (4178). District A recorded 2131 cases (vs 2246 in 2003) and District B 608 cases (vs 571 in 2003). Below we present evidence of direct and underlying influences on malaria persistence in both districts at health system levels (district, commune, village) and community level, in turn.

### District hospital level: satisfactory standards of malaria care but early discharge for some patients

Record review from the first 9 months of the year showed that DH-A treated 433 malaria cases. Review of a total of 88 patient cards from the two DHs showed close adherence to the most recent national malaria guidelines [17]. Just 3 patients were treated for malaria despite having a parasite-negative slide. Most DH malaria control staff were trained in the guidelines and generally knowledgeable. Of the 11 questions, the 8 staff at DH-A correctly answered all but 3, with 1–3 staff incorrect on each. Of the 6 DH-B respondents, all got 5 questions correct, with one wrong answer apiece for the remaining 6 questions. Microscopes were in good condition, microscopists had specialist training, and results were reportedly usually available within 30 minutes. There was one notable problem noted by DH staff during a community meeting: about one-third of inpatients discharged themselves prior to completion of treatment. Staff attributed this to inability to afford 'extra' charges for in-patient care, e.g. antibiotics and vitamins. Many were lost to follow-up, making it impossible to verify their adherence or recovery. However, most patients presented first to lower levels (though some were referred to DHs). At their last bout of malaria, 38% of community members reported they sought care from the VHW and 60% from the CHS; just 10% travelled to the DH (>one answer possible).

### Commune Health Stations: deficiencies linked to resources

Each commune in Vietnam has a health station in a fixed facility serving the commune's villages. National policy states CHSs should have at least 4 staff, including a fully-qualified doctor, nurses and/or midwives, and should implement all basic preventive and curative care under DH direction. Just 2 of our 4 communes had the full staff complement, but also had larger populations than usual. The others had 3 staff, though some were not qualified to offer routine services.

Checks found deficiencies at most CHSs in malaria diagnosis, treatment and microscopy. During FGDs and individual interviews, staff at all 4 communes acknowledged that presumptive treatment frequently occurred. A detailed record review for the first 9 months of 2004 was undertaken in one CHS (popn 2618) in District A; staff treated 100 parasite-positive and 82 'clinical' cases (unconfirmed by microscopy and diagnosed by symptoms). Thus nearly half of all cases (i.e. 82/182) were treated presumptively. Review of the past 3 months of logs in all 4 CHSs showed that in 2 communes, staff gave appropriate treatment per guidelines. In the other 2, staff sometimes gave CV8 for *P. vivax *cases (when chloroquine temporarily ran out) and primaquine + artesunate for clinical cases; moreover, workers at these CHSs did not recognise these treatments were contrary to guidelines. Laminated treatment guidelines intended for display to facilitate their use were locked out of sight in 3 of the 4 CHSs.

Although CHS staff discharged patients with instructions to report to their VHW during treatment, staff (at both levels) said patients often failed to do so, making it impossible to monitor adherence to treatment and course of illness, both of which are important for effective malaria control at the population level.

Several underlying influences apparently contributed to CHS-level weaknesses, including deficiencies in human resources, training, equipment and supply, all exacerbated by geographic isolation. In SSIs and FGDs most CHS staff said they found it difficult to accomplish their duties given current staffing levels. Understaffing placed particular pressures on microscopy services. Blood films would arrive haphazardly via VHWs or outpatient CHS services. Slides should be prepared and read immediately, which takes 30–45 minutes, but this rarely happened because of competing tasks, e.g. queues of infants awaiting immunisation, disease outbreaks, meetings with district health staff, or absence of the microscopist. For each slide the microscopist is paid an 'incentive' of just 300 dong (about USD two cents), which is low even by local standards. This situation may help explain why staff frequently prescribed anti-malarials according to symptoms, rather than after microscopic confirmation, as is preferred. For quality assurance, district staff periodically collected slides for review at the provincial capital; the percentage of incorrect readings was reported back to the district, and thence to each CHS, but without specifics on individual slides. One commune was told that 20% were incorrect after awaiting feedback for 4 months.

Although the MC program stipulates a properly trained microscopist for each CHS, most CHSs relied on one of their staff who was designated for this role but undertook the usual CHS workload, and typically had just a week of training. Few had in-service training. As well, quality was undermined by ageing microscopes, lack of stain solution in one commune, improper storage of materials in another, and inadequate pure water and filtering equipment in several.

The geographic features that make malaria viable in this region, coupled with low population density, present great challenges for its control. Poor roads, many waterways, steep ravines and a dearth of telephones hinder communications and transportation. Home visits, referrals and patient follow-up were particularly difficult, especially considering understaffing and (at the time of the study) lack of telephones in some CHSs, leading at times to local management of severe cases who would have been referred to the DH.

### Poorly trained Village Health Workers and lack of appropriate drugs

Among the 16 VHWs surveyed, most (14) were men, 12 were Van Kieu, 3 were Kinh (ethnic Vietnamese), and one Kazo. Median age was 31 years (range: 21–45 years). All had regular occupations as farmers (14) or traders (2). The 2 female VHWs had the highest education (10–12 years), 10 of the men had 6–9 years, and the other 4 had the minimum required (5 years) for VHWs. Median length of service was 5 years (range: 7 months-15 years).

The VHW (one per village) is a volunteer working across all primary health care programs following very basic training. For MC alone, VHWs are expected to prepare blood films, make referrals for severe cases, treat with (free) anti-malarials, educate the community, manage cases discharged from higher levels, and assist with spraying and net impregnation. The study found that some VHWs lacked confidence in their clinical MC duties (see Table 2).

**Table 2:** VHW self-reported confidence in aspects of malaria control role

	n (%)
Diagnose malaria through symptoms (n = 15*)	10 (66)
Take a blood sample from patients (n = 15*)	9 (60)
Make a blood film for microscopy (n = 15*)	9 (60)
Give correct anti-malarial medication (n = 15*)	9 (60)
Adequately manage malaria cases (n = 15*)	12 (80)
Undertake malaria information-education-communication activities (n = 16)	13 (81)

* missing data

KAP analysis revealed that 11/16 VHWs prepared blood films, but only 6 delivered these the same day to the CHS, with 4 waiting >72 hours. Ten said they 'rarely or never' stayed to obtain results; only parasite positive results were reported back to them from the CHS, often after a few more days. Most (11/16) commenced treatment without microscopic-confirmed diagnosis, prescribing partly by symptoms, and partly by the type of drug currently on hand within their kits. In 10 villages VHWs did not carry artesunate, the recommended drug of choice for *P. falciparum *malaria at the time of the study (see Table 3).

**Table 3:** Number of VHWs carrying different types of anti-malarial drugs

	**n = 16**
Number who carry only chloroquine	8
Number who carry only artesunate	0
Number who carry chloroquine & artesunate	6
Number who do not carry any anti-malarial drugs	2

Of the 6 who carried artesunate, all believed it was appropriate for 'serious' malaria cases. The main indication for chloroquine offered by the 14 who carried it was 'light' fever, not its usefulness for *P. vivax *malaria. Hence, use of anti-malarials for non-malarial fever may have occurred. Despite the fact that 12 VHWs reported confidence in case management, 8 admitted they never followed up.

Triangulation of data sets suggests that VHW weaknesses in malaria management were attributable to a number of underlying influences, including insufficient time to complete duties outside normal working hours, inadequacies in pre- and in-service training and some delays in rolling out the new guidelines for drugs in VHW kits.

In Vietnam, individuals often become VHWs out of civic duty or the appeal of further education and occasional – if small – incentives for particular health care tasks. Apart from their MC duties, VHWs must keep abreast of changing, relatively complex, treatment guidelines. This is daunting for volunteers with low education levels residing in remote locations. When asked to name the role's disadvantages, our sample mentioned low remuneration, lack of time, and difficulties with transportation and distance, all of which could undermine case identification and management. About one-third felt frustrated by the villagers' 'refusal to take advice'.

Although policy dictates that each VHW is trained pre-service for at least three months, just 4 (one-quarter) had such training; 3 had 12–45 days, 6 had 1–5 days, and 3 reported no training. Only 5 reported training during 2004, although provincial policy requires annual refresher training. Only 12 VHWs knew about the new guidelines and 10 carried the new treatment table. Most, however, knew correct dosage for the drugs they carried. At the time of the study NIMPE was disseminating new diagnosis and treatment guidelines, which include some devolution of decision-making on local treatment to provincial MC managers. Some confusion appeared to persist during this transition, because informants at various levels provided inconsistent information about policy for anti-malarials in VHW drug kits, and a range of explanations for what was actually in the kits.

The terrain and isolation that hinder optimal care by CHSs act as greater barriers for the VHW MC role, because VHWs typically have even less access to reliable transportation. It takes time, effort and – at the least – opportunity costs for these part-time volunteers to remain in close touch with higher health system levels, to follow up or to refer patients. These circumstances presented ongoing risks that some seriously ill patients would be treated in the village, possibly with a less effective anti-malarial.

### Community level: sub-optimal prevention linked to insufficient bed-nets and socio-cultural context

Demographic information from the community KAP appears in Table 4. Most were Van Kieu, and education levels were low, with females more likely to be unschooled (χ^2 ^= 28.22, p = 0.01). Median household size was 6 persons (range 2–13 persons). Sixty percent had a 'Poor Card', which denotes low-income status and enables free medical care and basic drugs. Most (66%) survey respondents reported having had malaria, including about one-third at least once in 2004.

**Table 4:** Description of the KAP community sample, by sex

	**Males** **n (%)**	**Females** **n (%)**	**Total** **n (%)**
Sex	80 (50)	80 (50)	160 (100)

Age (mean, range) in years	34, 18–48	30.6, 18–45	32.5, 18–48

Ethnicity:			
Van Kieu	69 (86)	71 (89)	140 (87.5)
Kinh (Vietnamese)	8 (10)	9 (11)	17 (10.6)
Other	3 (4)	0	3 (1.9)

Education level reached:			
No schooling	31 (39)	64 (80)	95 (59.3)
Some primary (1–5 years)	27 (34)	4 (5)	31 (19.3)
Some secondary (6–9 years)	22 (27)	12 (15)	34 (21.3)

Occupation			
Farmer	67 (84)	68 (85)	135 (84.3)
Other	11 (14)	9 (11)	20 (12.5)
missing	2 (2)	3 (4)	5 (3.1)

Poor Card			
Yes	44 (55)	52 (65)	96 (60)
No	35 (44)	27 (34)	62 (38.8)
missing	1 (1)	1 (1)	2 (1.3)

Our findings suggest the direct risks operating at community level were sub-optimal bed-net use and early self-discharge from care. The national MC program calculates net sufficiency on a ratio of one net per two people, with a target of consistent use by at least 80% of the population in endemic areas. Quang Tri health staff at all levels believed this target was not met in the study communes, a view based on irregular day-time spot checking by provincial and district survey teams. We undertook our estimates differently, i.e. by observation during unannounced night-time visits, coupled with survey questions on bed-net use. Night visits to 55 homes in two communes found no nets were used in 20% of households and some nets did not reach the floor or were used as blankets. The 160 survey respondents, however, reported very high usage: 145 (92%) claimed to have slept under a net on the previous night, and 136 (86%) said that all family members had done so, whether singly or (more frequently) sharing. Respondents cited adolescents and the elderly as less likely to use and/or share nets, with just 50% of teenagers consistently using, among whom 70% shared. Reportedly, 91% of elders 'always' used nets, but only 57% shared. Whilst 16% of respondents claimed to travel occasionally or often into Laos, and about half went into forests at varying frequency, just a handful carried bed-nets on overnight trips.

While 66 (41%) sometimes (n = 58) or always (n = 8) consulted traditional healers for 'health problems', the survey showed high awareness of recommended help-seeking for suspected malaria. Respondents claimed to act accordingly (Table 5), although this could not be verified. Some malaria patients with Poor Cards said they were charged for extras like vitamins at DHs (6/20) and CHSs (12/113), leading some to borrow money or discharge themselves early.

**Table 5:** Community responses about care-seeking for suspected malaria

What to do first for fever or suspected malaria (n = 149*)	**n (%)**
Do nothing	1 (0.7)
Pray	3 (2.0)
Buy drug in market	4 (2.7)
Go to Village Health Worker	77 (52)
Go to Commune Health Station	63 (42)
Go to District Hospital	1 (0.7)

How long do you wait before seeking care? (n = 129*)	

Immediately	81 (63)
One day	41 (32)
Two days	6 (4.7)
More than two days	1 (0.8)

* Missing data

There was considerable evidence that insufficient bed-nets, cultural sleeping norms, low education and poverty acted as underlying influences on sub-optimal community behaviours. Provincial staff told us that Quang Tri had comprehensive bed-net coverage through the NMCP, and MC staff at all levels attributed persistent malaria in Quang Tri mainly to community 'refusal' to use bed-nets, arguing the need for more 'information, education and communication'. While enough nets may have been distributed, our survey respondents reportedly received theirs prior to 2003, and many were no longer intact. Some purchased additional nets, usually cheaper single bed size. Using MC guidelines on bed-net ratios (one net/2 people) and data on household size, we calculated that among the 160 households represented by survey respondents, just 72 (45%) had sufficient nets to cover their needs and 88 (55%) did not. In addition, checks of net quality when conducting the survey found 62% of households had at least one ripped or damaged net. Thus, even if all available nets were used, *less than half of all households were fully protected*.

Family configuration and cultural sleeping patterns also affected net adequacy. In FGDs we heard that some teenagers refuse to use nets, and that elders (with reportedly high net usage) strongly prefer to sleep alone, thus potentially leaving other family members short. As well, overnight socialising among male neighbours is so normal that Van Kieu houses contain a nominated 'guest' space in the living room, but just 19% of respondents had a spare net for guests.

A lack of spare nets also contributes to exposure risk during periods of mobility – usually by foot – into Laos, forest or fields for overnight stays. This mobility is culturally and economically driven, as families seek reunions with relatives across the Lao border, and individuals collect forest products for consumption or sale due to lack of employment options.

As Table 6 illustrates, most respondents had basic understanding of malaria symptoms and causation, and knew malaria is curable. However, about one-quarter were unsure about causation and prevention. Among those who said malaria is not preventable, 28 (55%) had no schooling, versus just 5 (18%) with one or more years of schooling (χ^2 ^14.33, p = 0.001); this misperception was held by 17.5% of men and 50% of women(χ^2 ^6.60, p = 0.01). The lower education levels of women in particular may explain gaps in preventive behaviours.

**Table 6:** Community knowledge about malaria transmission, prevention and cure

	n (%)
Have heard of malaria (n = 158*)	143 (91)
Mosquitoes main 'cause' of malaria (n = 158*)	113 (72)
'Don't know' what causes malaria (n = 158*)	40 (25)
Fever is a symptom of malaria (n = 160)	124 (77)
Malaria can be cured (n = 156*)	134 (84)
Malaria can be prevented (n = 140*)	107 (76)
Bed-net is best way to prevent malaria (n = 160)	98 (61)
'Don't know' best way to prevent malaria (n = 160)	53 (33)

* missing data

Ethnic minorities in western Quang Tri have little involvement with mainstream society. Whilst VHWs tend to be the same ethnicity as villagers, this is less true for other providers. A third of respondents 'sometimes' had language problems with district or commune providers, and one ethnic Vietnamese commune health worker who spoke Van Kieu felt neither fully accepted nor fully trusted.

In theory, cost should not deter care-seeking because malaria diagnosis and treatment are free. However, these involve transportation, opportunity and (sometimes) medical 'extras' costs that this community could ill-afford, which may help explain why some discharged themselves from care and were lost to follow-up. Such charges are imposed increasingly as Vietnam's health system is decentralised.

## Discussion

This mixed-method study in Quang Tri province in central Vietnam was designed by a multi-disciplinary team that included malaria experts and social scientists. It set out to map the non-biological 'causal pathways' that led to the problem of persistent malaria in a remote ethnic minority population. As Hawe et al argue, exploring the underlying influences that precipitate, amplify or mitigate direct health risks provides evidence that can assist programmers to design and target comprehensive interventions to bring about and sustain necessary changes; the same approach used in program evaluation can pinpoint specific opportunities to address quality concerns [18].

### Strengths and weaknesses of the study

Particular strengths of the study were the involvement of stakeholders from various health levels, including the community itself, and the triangulation of data through use of multiple methods (quantitative and qualitative), including self-report and the more objective tools of observation and record review. Malaria social scientists have noted the need for community-level malaria investigations to commence with qualitative methods that help explain behaviours, thus permitting grounded development of structured surveys [19]. This formative approach was one of our study's strengths. However, due to lack of resources and expertise, systematic preparation and analysis of complete transcripts were not conducted, preventing full utilisation of qualitative data to illuminate the study's quantitative findings.

Another limitation was a lack of definitive data from CHWs on case management and microscopy quality, which reflects the more rudimentary health reporting often found in remote settings. However, our objective was to map pathways in one study site and not to produce generalisable findings, which in any case would be inappropriate given the small number of communes explored and relatively small sample of providers and community members. This study also did not attempt to identify the role of biological factors such as vector prevalence or drug sensitivity; thus preventing us from arguing conclusively the relative importance of all potential factors.

### Systems and the community: a dual focus for malaria control in remote settings

**Figure 1:**
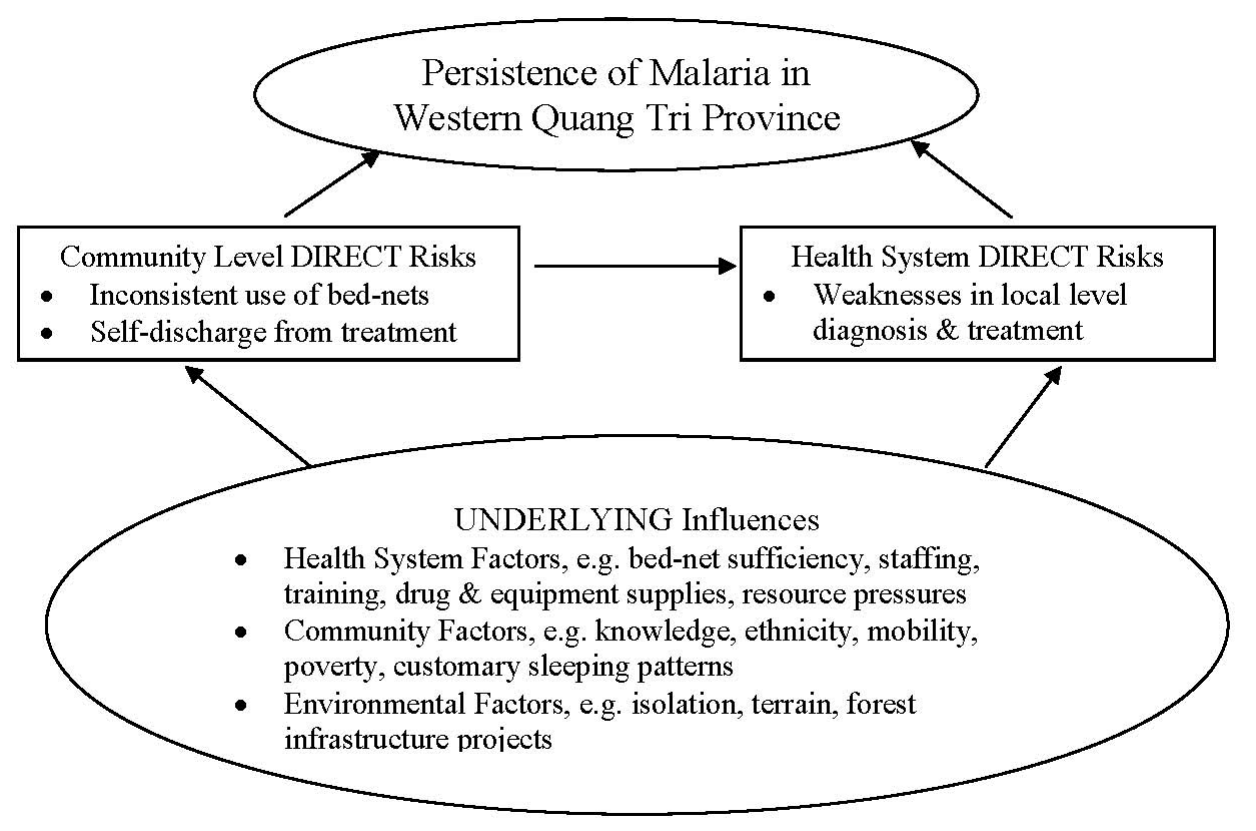
**Model of non-biological pathways to malaria persistence in Quang Tri province**.

Figure 1 summarises relationships and pathways to malaria persistence drawn from this study and lays out the underlying influences that apparently explained weaknesses found at both health systems and community levels. This model excludes vectors, weather events and drug sensitivity. We present this as a conceptual framework for mapping our findings, and for possible adaption by researchers wishing to investigate such pathways in other complex settings.

Previous studies in Vietnam have found widespread misunderstanding about malaria treatment and prevention among populations in similar isolated endemic areas [20,21]. Our study found around a quarter of the community shared these misunderstandings, and our model suggests this may have contributed to poor health behaviours. Health systems managers often assume (as here) that minority group customs, culture or knowledge 'barriers' account for poor behaviours (and outcomes), assumptions that typically lead solely to community education interventions. The national program's ratio for bed-net sufficiency also rests upon assumptions about net-sharing, and about where people actually sleep. Our major finding – that over half of households surveyed lacked sufficient bed-nets – illustrates the risks of untested assumptions, particularly in view of population sleeping patterns and mobility through forests and borders, which increases net requirements while enhancing exposure risk. A recent study in Vietnam found that regular forest work accounted for 53% of *P. falciparum *infections, with increased risk if people used nets at home but not in forests [22]. Another found that movement of infrastructure project workers within forests (which was occurring in our site) was a source of ongoing malaria [4]. While respondents – particularly women and the unschooled – require an appropriate educational program, it is clear that responsibility for non-use of bed-nets, and/or ongoing malaria, cannot fully be placed at the feet of this community.

A review by Williams and Jones [23] found that malaria studies typically focused on the role of mothers or care givers in malaria management, while few looked at health care quality. This is surprising given the pivotal role played by both providers and rational drug use. The World Health Organization [24] has noted that health worker shortages – an increasing global problem and one found in our site – are linked to higher mortality rates. A recent review [25] of the impact of health reforms on Vietnam's commune-level services found poorer quality CHSs in remote areas, especially where ethnic minorities live. We found that local providers often lacked diagnostic skills, time, equipment and/or appropriate drugs for populations in this remote region. Even temporary shortfalls in the supply of anti-malarial drugs, especially during outbreaks, could have serious impacts. Additionally, District Hospital staff estimated that one-third of malaria patients discharged themselves early for cost reasons (medical 'extras'), and were usually lost to follow-up. Thus, presumptive, under-treatment and unnecessary treatment probably occurred, which are known to endanger individual patients and may contribute to the emergence of drug resistance [1].

## Conclusion

A recent multi-country analysis found increasing use of income-generating malaria services and reductions in free services, with low provider salaries associated with inappropriate care-giving [26]. Regional disparities in revenue-raising and human resources can result in uneven implementation of control programs [27]. In a poor province with limited revenues like Quang Tri, care must be taken to ensure that pressures to charge additional service fees do not discourage people from seeking and completing malaria treatment. Malaria control in this site cannot be achieved through community education alone. Focused training, strategies to attract staff to remote areas, appropriate transportation and communication systems, greater efforts to keep (often impoverished) patients under care, and robust supply chains for drugs and impregnated bed-nets – with regular monitoring of use, quality and sufficiency – are among the responses that can further strengthen Vietnam's efforts to address malaria persistence in this isolated region.

## Abbreviations

CHS: Commune Health Station; DH: District Hospital; FGD: Focus Group Discussion; KAP: Knowledge, Attitudes and Practices; MC: Malaria Control; NMCP: National Malaria Control Program; NIMPE: National Institute for Malariology, Parasitology and Entomology; SSI: Semi-structuredInterview; VHW: Village Health Worker.

## Competing interests

The authors declare that they have no competing interests.

## Authors' contributions

MM conceptualised and designed the study, trained co-investigators, led the analysis process and was primarily responsible for drafting the manuscript. QAN coordinated the field work and conducted the majority of field research, entered and analysed quantitative data and contributed to the analysis process. SC made substantial contributions to training of co-investigators, data analysis and revision of manuscript drafts. BAB contributed to the analysis process and revision of the manuscript. NHD and TTN contributed to analysis of data and revision of the manuscript. All authors read and approved the final manuscript.

## Pre-publication history

The pre-publication history for this paper can be accessed here:


